# The role of *N-glycans* in colorectal cancer progression: potential biomarkers and therapeutic applications

**DOI:** 10.18632/oncotarget.6283

**Published:** 2015-11-02

**Authors:** Julio Cesar Madureira de Freitas Junior, José Andrés Morgado-Díaz

**Affiliations:** ^1^ Cellular Biology Program, Structural Biology Group, Brazilian National Cancer Institute (INCA), Rio de Janeiro, RJ, Brazil

**Keywords:** N-glycans, colorectal cancer, biomarkers, glycosylation, carcinoma

## Abstract

Changes in glycosylation, which is one of the most common protein post-translational modifications, are considered to be a hallmark of cancer. *N*-glycans can modulate cell migration, cell-cell adhesion, cell signaling, growth and metastasis. The colorectal cancer (CRC) is a leading cause of cancer-related mortality and the correlation between CRC progression and changes in the pattern of expression of *N*-glycans is being considered in the search for new biomarkers. Here, we review the role of *N*-glycans in CRC cell biology. The perspectives on emerging *N*-glycan-related anticancer therapies, along with new insights and challenges, are also discussed.

## INTRODUCTION

### Colorectal cancer: epidemiology and clinicopathological features

Colorectal cancer (CRC) represents the third most commonly diagnosed cancer in males and the second most commonly diagnosed cancer in females. Over 1.3 million new cancer cases and 693,881 deaths were estimated to have occurred worldwide in 2012 [1]. CRC is a leading cause of cancer-related mortality, and it accounts for 8.5% of all cancer-related deaths. The 5-year survival rate depends on the stage and reaches up to 6% for stage IV [2]. The cumulative lifetime risk for CRC is 5-6%; this risk is influenced by hereditary and lifestyle factors [3]. Most CRC cases are the sporadic form of the disease; however, 20-30% of cases are hereditary or familial [4]. Well-defined high-risk CRC syndromes, such as familial adenomatous polyposis (FAP) and hereditary nonpolyposis colon cancer (HNPCC), account for 3-5% of all colon cancer cases [5, 6]. The etiologies of the remaining inherited CRCs are not completely understood. Several lifestyle risk factors have been related to the development of CRC. For instance, heavy alcohol intake and obesity appear to increase the risk, whereas the consumption of fruits, vegetables, and a high-fiber diet may be protective against CRC [7, 8]. Additionally, an association between cigarette smoking and *APC* mutations, which are the most common mutations in CRC, has been shown [9].

**Figure 1:**
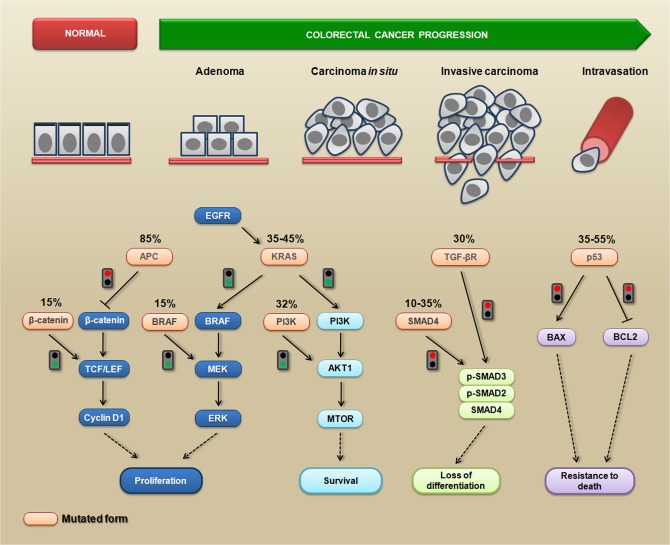
The most common genetic mutations during CRC progression and the triggered cellular mechanisms Colorectal cancer progression is a multistep process during which several phenotypic changes occur, leading to enhanced proliferative abilities, the activation of survival signaling, the loss of differentiation status, resistance to death, and the acquisition of invasive and migratory abilities. Mutated APC is unable (red signal) to inhibit the accumulation of cytoplasmic β-catenin; consequently, it translocates to the nucleus where it promotes the activation of transcription factors from the TCF/LEF family, leading to proliferation via enhancing cyclin D1 levels. Alternatively, the same pathway can become constitutively active when β-catenin is mutated (green signal). The expression of a constitutively active KRAS mutant triggers (green signal) downstream pro-proliferative signaling (BRAF/MEK/ERK) and pro-survival signaling (PI3K/AKT1/MTOR). This signaling can also be triggered by mutations in BRAF and PI3K (green signals). Mutations in SMAD4 or TGF-β (red signals) disrupt the TGF-β-induced formation of the SMAD2/3/4 complex, which contributes to the loss of differentiation. The mutated p53 protein is unable (red signal) to enhance the levels of the pro-apoptotic BAX protein, preventing cytochrome-c release from the mitochondria. This mutated form is also unable (red signal) to inhibit the anti-apoptotic BCL2 protein, leading to a death resistance phenotype.

CRC progression occurs through a series of well-defined clinical and histopathological stages, mostly ranging from single crypt lesions through small benign tumors (adenomatous polyps) to malignant cancers (carcinomas) [10]. The different histopathological stages reflect the accumulation of genetic changes that occur during tumor progression, which is caused by genomic instability [11, 12]. These genetic changes affect the functionality of several signaling-related proteins that are involved in cell proliferation, survival, migration and invasion [13]. For example, *APC* mutations directly affect β-catenin (a dual-function protein involved in cell-cell adhesion and signaling) levels, leading to the transcriptional activation of the TCF/LEF target genes, which can induce cell proliferation and inhibit cell-cell adhesion [14, 15]. The RAS-RAF-MAPK and PI3K-AKT1-MTOR pathways are frequently affected by oncogenic alterations, and these pathways are strictly interconnected to modulate several cellular mechanisms related to the malignant phenotype, such as cell proliferation, survival, and migration [16]. More importantly, *SMAD4* mutations can lead to the loss of both the differentiation and growth inhibitory effects mediated by TGF-β [17]. Additionally, oncogenic mutations in *TP53* result in the inactivation of p53-dependent proapoptotic signaling [18]. It is important to note that the *APC* mutation is the first mutation in the majority of CRCs, but the precise order of the subsequent mutations may vary from tumor to tumor. Moreover, some mutations, such as *KRAS* and *BRAF*, appear to occur in a mutually exclusive fashion [19]. Figure 1 summarizes the most common CRC-related genetic mutations and the triggered cellular response.

In addition to these well-known genetic changes, which promote malignant behavior, post-translational modifications regulate molecular mechanisms contributing to phenotypic changes in cancer cells. One of these post-translational modifications is the protein glycosylation, however, the molecular details and the role that this regulatory mechanism play during CRC progression is not yet completely understood and will be discussed here.

### Glycosylation and *N*-glycans

Glycosylation is one of the most common protein post-translational modifications. Recent data estimate that approximately one-fifth of all proteins are glycosylated [20]. Protein glycosylation occurs primarily in the endoplasmic reticulum (ER) and the Golgi apparatus and involves a complex series of enzymatic reactions catalyzed by glycosyltransferases and glycosidases. An increasing number of studies in the last decades have focused on investigating the functional roles of carbohydrate structures, opening a field in life science denominated as functional glycomics.

**Figure 2:**
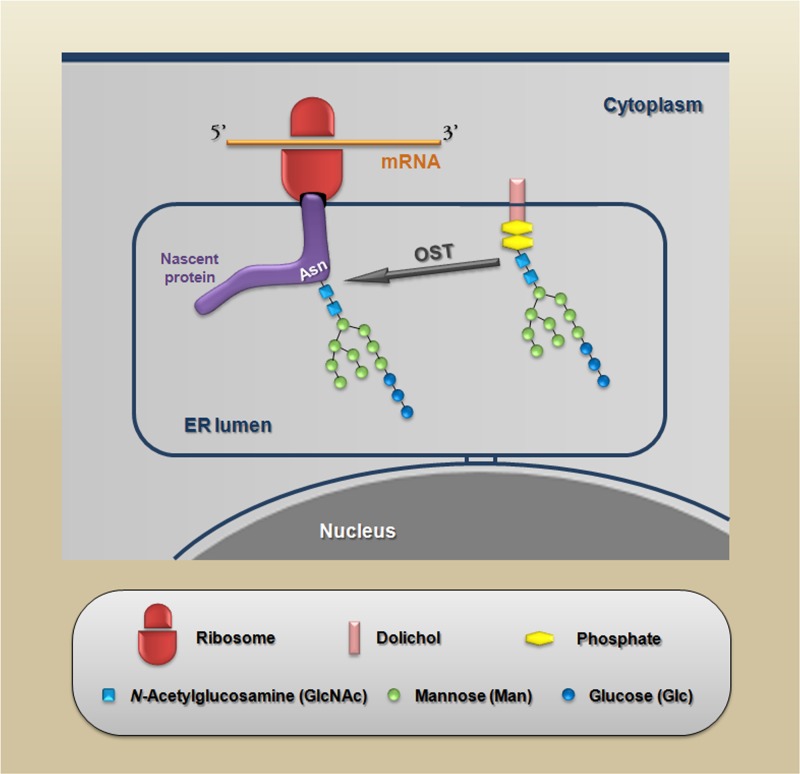
Schematic representation of the protein *N*-glycosylation reaction The nascent proteins synthesized within the endoplasmic reticulum are glycosylated via the *en bloc* transfer of a 14-sugar compound to an asparagine residue in a specific consensus sequence (Asn-X-Ser/Thr) in which X is any amino acid except proline. This reaction is catalyzed by OST (oligosaccharyltransferase).

**Figure 3:**
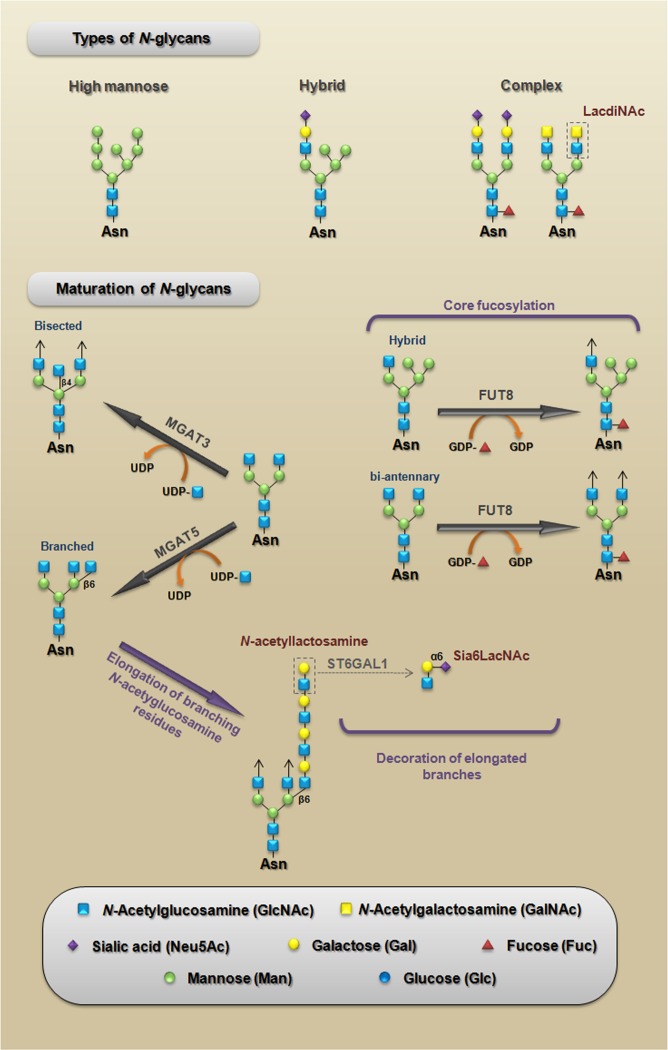
Types of *N*-glycans A schematic representation of the three main structures that are synthesized during *N*-glycan processing (upper panel). **Maturation of N-glycans**. A schematic representation of the three components of maturation of *N*-glycans: (I) core fucosylation representing the addition of monosaccharides to the core; (II) *N*-acetyllactosamine structure, which represents the elongation of branching *N*-acetylglucosamine residues; and (III) the synthesis of Sia6LacNAc, which represents the decoration of elongated branches (lower panel). LacdiNAc, *N*-acetyllactosamine, and Sia6LacNAc are cancer-associated carbohydrate antigens whose functional roles during CRC will be discussed in this paper. MGAT5, *N*-acetylglucosaminyltransferase V [mannosyl (alpha-1,6-)-glycoprotein beta-1,6-N-acetyl-glucosaminyltransferase]; MGAT3, *N*-acetylglucosaminyltransferase III [mannosyl (beta-1,4-)-glycoprotein beta-1,4-N-acetylglucosaminyltransferase]; FUT8, α1,6-fucosyltransferase [fucosyltransferase 8 (alpha (1,6) fucosyltransferase)]; ST6GAL1, α2,6-sialyltransferase [ST6 beta-galactosamide alpha-2,6-sialyltranferase 1].

Many of the carbohydrate-mediated cellular mechanisms, including those with important implications for tumor progression, are regulated by *N*-glycans. *N*-glycosylation is initiated by the synthesis of a dolichol lipid-linked oligosaccharide precursor, which is transferred *en bloc* to the asparagine residues (Asn-X-Ser/Thr motif) of nascent proteins. This reaction is catalyzed by the oligosaccharyltransferase (OST) enzyme complex in the ER (Figure 2). Then, the oligosaccharide processing includes the addition and removal of monosaccharides; these reactions are catalyzed by glycosyltransferases and glycosidases, respectively. During this processing three main structures are synthesized: (I) the high-mannose type (synthesized in the ER) which represents the early stage of processing; (II) the hybrid type (synthesized in the Golgi apparatus), which shows both high-mannose and complex-type features; and (III) the complex type (synthesized in the Golgi apparatus), in which the addition of *N*-acetylglucosamine (GlcNAc) allows the elongation of the chains, which are referred to as antennae (bi-, tri- or tetra-antennary structures) (Figure 3 upper panel).

During the maturation process in the Golgi, a limited repertoire of hybrid and branched structures are converted into a large quantity of mature, complex *N*-glycan. This process can be divided into three components: (I) the addition of monosaccharides to the core; (II) the capping or elongation of branching *N*-acetylglucosamine residues; and (III) the decoration of elongated branches (Figure 3, lower panel). It is important to note that β1,6-branched, bisected (β1,4 GlcNAc) and core fucosylated (α1,6 fucose) *N*-glycans are enzymatic products of *N*-acetylglucosaminyltransferase V (MGAT5), *N*-acetylglucosaminyltransferase III (MGAT3) and α1,6-Fucosyltransferase (FUT8), respectively; in epithelia, changes in the expression patterns of these *N*-glycans have been associated with several pathologies, including carcinomas [21]. Other structures that are generated during the elongation of *N*-acetylglucosamine residues, such as LacdiNAc termini and α2,6-sialylated lactosamine (Sia6LacNAc), have been also considered to be cancer-associated carbohydrate antigens (Figure 3 upper and lower panel). The levels of cancer-associated carbohydrate antigens are regulated by several mechanisms; for example, they can be modulated by nucleotide sugar donor availability (e.g., UDP-GlcNAc and GDP-Fuc levels) or by changes in the expression levels of both glycosyltransferases and glycosidases [22, 23].

Changes in glycosylation are considered to be a hallmark of cancer. *N*-glycans participate in several cellular mechanisms, such as metabolism, signaling, growth, cell-cell adhesion, cell-matrix interaction, invasion, and metastasis. Next, we discuss the involvement of *N*-glycans in some of these fundamental biological processes involved in cancer in a general manner and then we discuss the data regarding CRC specifically. We apologize to the authors whose important contributions could not be cited because of space limitations.

### *N*-glycans and the fundamental biological processes involved in cancer

An important question in cancer biology is how changes in glycan expression are related to carcinoma progression. In many cases is not yet clear whether changes in the expression of *N*-glycans is a con-cause, or a consequence of cancer progression (e.g., reciprocal regulatory processes). So, hereafter, we will emphasize these cause-effect relationships when necessary.

#### *N*-glycans and the cancer-related metabolic/signaling processes

**Figure 4:**
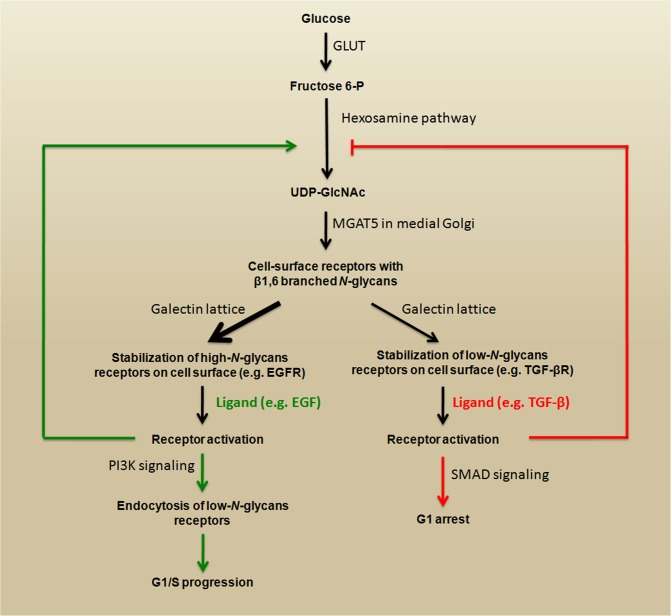
Regulation of the cell cycle in cancer by metabolic/signaling mechanism High levels of intracellular fructose 6-P fuel the hexosamine pathway by generating substrates (UDP-GlcNAc) for MGAT5. The products (β1,6 branched *N*-glycans) interact with galectins, which promote stabilization growth factor receptors on the cell surface. The stimulation of high-*N*-glycan or low-*N*-glycan receptors triggers opposite signaling that promotes G1/S progression or G1 arrest, respectively. This schematic representation is adapted from [27].

Some studies have shown a link between signaling pathways that play key roles during carcinoma progression and the expression of β1,6-branched *N*-glycans. The activation of phosphoinositide 3-kinase (PI3K) pro-survival signaling (see Figure 1) increases Golgi *N*-glycan processing, potentially by increasing MGAT5 expression, which could promote the stabilization of growth factor receptors on the cell surface as a result of increasing β1,6-branched structures. Therefore, MGAT5 and phosphatase and tensin homolog (PTEN) - a negative regulator of PI3K signaling - could interact in an opposing manner to regulate cellular sensitivities to extracellular growth cues [24]. A very important mechanism through which *N*-glycan branching cooperate to regulate cell proliferation and differentiation is the differential modulation of cell membrane receptors that present few or high numbers of *N*-glycans. The Golgi apparatus is very sensitive to hexosamine (six carbon sugars with an amino group, which is commonly *N*-acetylated, e.g., GlcNAc and GalNAc) fluxes for the production of tri- and tetra-antennary *N*-glycans, because the UDP-GlcNAc concentration is a crucial factor in the biosynthesis of β1,6-branched *N*-glycans (see Figure 3, lower panel) [22]. These tri- and tetra-antennary *N*-glycans bind to galectins (a group of proteins that bind to β-galactose-containing glycoconjugates) and form a galectin-3-dependent molecular lattice that opposes glycoprotein endocytosis [25, 26]. Thus, increasing intracellular UDP-GlcNAc enhances both branching and the association of membrane receptors (e.g., EGFR or TGF-βR) with galectin-3, promoting its stabilization at the cell surface and leading to increased signaling. However, with increased UDP-GlcNAc, the signaling response to EGF or TGF-β varies because the activation kinetics are hyperbolic for the growth-promoting receptors such as EGFR, PDGFR, IGFR, and FGFR (which have 8-16 *N*-glycosylation sites) and switch-like for TGF-βR (which has 1 or 2 *N*-glycosylation sites). Therefore, the cellular transition between growth and arrest appears to be regulated by metabolic/signaling mechanisms through nutrient flux (hexosamine), which is stimulated by growth-promoting high-*N*-glycan receptors (positive feedback via stabilization through galectin-branching interactions) to drive arrest/differentiation programs (a inhibitory feedback) by increasing the surface levels of low-*N*-glycan receptors [27, 28]. Altogether, these findings demonstrate that both N-glycan number and degree of branching orchestrate the regulation of cell proliferation and differentiation. Figure 4 summarizes a proposed model for regulation of the cell cycle in cancer by this metabolic/signaling mechanism.

#### *N*-glycans and the regulation of cell-cell adhesion and cell-matrix interactions

Carcinoma progression involves the loss of cell-cell adhesion and the acquisition of migratory and invasive potentials [29]. These events characterize the epithelial-mesenchymal transition (EMT), which is an important phenomenon that occurs during the progression of epithelial cancer to metastasis. In this context, many researchers have undertaken studies to understand the molecular mechanisms that regulate the EMT, including those related to changes in the expression of *N*-glycans.

E-cadherin, the main cell-cell adhesion molecule in epithelia, plays pivotal roles in the suppression of tumor cell migration and metastasis and is also a key molecular player in the EMT process [30]. Several mechanisms such as post-translational modifications by *N*-glycosylation have been recently proposed that underlie E-cadherin downregulation or inactivation in cancer, [31-35]. Human E-cadherin has four potential *N*-glycosylation sites [36], and several studies have corroborated the hypothesis that different expression profiles of E-cadherin-linked *N*-glycans are related to the stability of adherens junctions (AJs); therefore, alterations of these *N*-glycans play a crucial role in the acquisition of the malignant phenotype [37, 38]. On the one hand, increased E-cadherin modification with β1,6-branched *N*-glycans, which is catalyzed by MGAT5 [38, 39], is able to induce a destabilization of E-cadherin-mediated cell-cell adhesion with consequences to tumor progression [40]. On the other hand, the modification of E-cadherin with bisected *N*-glycans, which is catalyzed by MGAT3, increases the stability of AJs and is associated with the suppression of tumor progression [40, 41]. Recently, we demonstrated that the interplay between E-cadherin and IR/IGF-IR signaling modulates bisected *N*-glycan expression levels and consequently the invasive phenotype. The exogenous E-cadherin expression in MDA-MB-435 cells (which endogenously lack E-cadherin expression at both the mRNA and protein levels) inhibits IR, IGF-IR and downstream ERK 1/2 phosphorylation, whereas stimulation of MDA-MB-435+E-cad cells with insulin and IGF-I decreases the bisected *N*-glycan expression (in general and specifically on E-cadherin), up-regulates mesenchymal markers and enhances tumor cell invasion [42].

The modulation of the cell-matrix interaction and, consequently, the migratory potential, are also associated with changes in *N*-glycan expression. The biological functions of integrins (proteins that link the cells with the extracellular matrix) can be modified by the presence of different glycan patterns on these molecules [43]. Integrin α3β1-mediated cell migration on the laminin 5 substrate is greatly enhanced after the overexpression of MGAT5 in gastric cancer cells. Conversely, the level of cell migration in these cells is reduced after the overexpression of MGAT3 [23]. Considering that the products of MGAT3 are bisected *N*-glycans and the products of MGAT5 become tri- and tetra-antennary *N*-glycans, there is a hypothesis that these β1,6-branched molecules may act as a chemical barrier not only against the establishment of a stable cell-cell adhesion but also against the establishment of a stable cell-matrix interaction [44].

It is important to highlight that the role of β1,6-branched *N*-glycans in carcinoma cell migration does not to appear to be the same for all types of cancers. For example, in non-small-cell lung cancer cells, the inhibition or silencing of MGAT5 promotes TGF-β1-induced cell migration [45]. Nonetheless, the observations in gastric cancer cells strongly corroborate the hypothesis of the pro-migratory role of β1,6-branched *N*-glycans during carcinoma progression.

#### *N*-glycans and cell growth, invasion, and metastasis

Changes in the expression patterns of β1,6-branched and bisected *N*-glycans have also been associated with increased replicative potential, tissue invasion, and metastasis, which constitute hallmarks of cancer progression [46]. For example, mammary tumor growth and metastasis induced by the polyomavirus middle T (PyMT) oncogene are reduced in *Mgat5*^−/−^ mice (knockout for the gene encoding MGAT5) [47], whereas *Mgat3*^−/−^ mice (knockout for the gene encoding MGAT3) exhibit an accelerated appearance of mammary tumors induced by PyMT [48]. Paradoxically, enhanced expression of MGAT3 has been reported in rat hepatomas, but the progression of hepatic neoplasms is severely retarded in mice lacking bisected *N*-glycans [49, 50]. These studies show that although MGAT3 apparently affects cancer development, the effects triggered by the increase of bisected *N*-glycan expression could be tissue/organ specific.

In gastric cancer cells, the overexpression of MGAT5 leads to a severe peritoneal dissemination of tumor cells in athymic mice, which was attributed to the prolonged stabilization of matriptase. Matriptase is a tumor-associated type II transmembrane serine protease that is positively regulated during metastasis by activation of the latent forms of hepatocyte growth factor (HGF) and urokinase-type plasminogen activator (uPA) [51, 52]. Immunohistochemical analyses of surgically resected samples showed that high expression of MGAT5 appears to be involved in the malignant potential (lymph vascular space involvement) of endometrial and mucinous ovarian cancer [53, 54], but high levels of MGAT5 are also related to a low malignant potential and good prognosis for patients with bladder cancer [55]. Again, these results show that, similar to those observed for bisected *N*-glycans, the effects triggered by β1,6-branched *N*-glycan expression could also be tissue/organ specific.

In this second part of review, we specifically discuss the recent progress regarding the role played by different *N*-glycans in CRC. We also discuss *N*-glycans as new potential biomarkers and their future therapeutic applications.

## THE ROLE OF N-GLYCANS IN CRC PROGRESSION

Studies have shown that changes in the expression of enzymes involved in *N*-glycan biosynthesis and their products can modulate cell-cell adhesion, cell signaling, stemness and invasiveness in CRC cells.

### *N*-glycosylation degree modulates E-cadherin-mediated cell-cell adhesion

According the length of *N*-glycans (e.g., degree of branching) or the number of *N*-glycosylation sites (Asn-X-Ser/Thr motif) occupied by *N*-glycans, glycoproteins can be classified as hyper- or hypoglycosylated and their biological functions can be modified by the presence of these different glycan patterns. In this context, we have previously demonstrated that E-cadherin from human colon carcinoma HCT-116 cells, which have unstable AJs, is more richly *N*-glycosylated than E-cadherin from Caco-2 cells, which have stable AJs, in addition, the inhibition of *N*-glycan biosynthesis induces a functional E-cadherin-mediated cell-cell adhesion in HCT-116, which was disrupted by calcium depletion [37]. A key regulator of protein *N*-glycosylation is the *DPAGT1* gene, which encodes the dolichol-P-dependent *N*-acetylglucosamine-1-phosphate transferase; this enzyme initiates the synthesis of the dolichol lipid-linked oligosaccharide precursor (see structure in Figure 2) for protein *N*-glycosylation in the endoplasmic reticulum (ER). Interestingly, *DPAGT1* is a target of the canonical Wnt/β-catenin signaling pathway [83,87], which is commonly affected in CRC cells, thus reinforcing the idea that disruption of E-cadherin-mediated cell-cell adhesion by increased *N*-glycosylation may be important during the progression of CRC.

Additionally, core fucosylation of E-cadherin-linked *N*-glycans modulate its function in CRC cells. The transfer of a fucose residue from GDP-fuc to position 6 of the innermost GlcNAc residue of *N*-glycans is catalyzed by FUT8 and produces core fucosylated glycoproteins (see Figure 3). Interestingly, overexpression of FUT8 reduces the turnover rate of E-cadherin, and core fucosylation of their *N*-glycans enhances cell-cell adhesion in human colon carcinoma WiDr cells [56].

### β1,6-branched and bisected *N*-glycans modulate cell behavior

#### Reciprocal regulation of MGAT3 and adherens junctions and the involvement of Wnt/β-catenin signaling in MGAT3 expression

**Figure 5:**
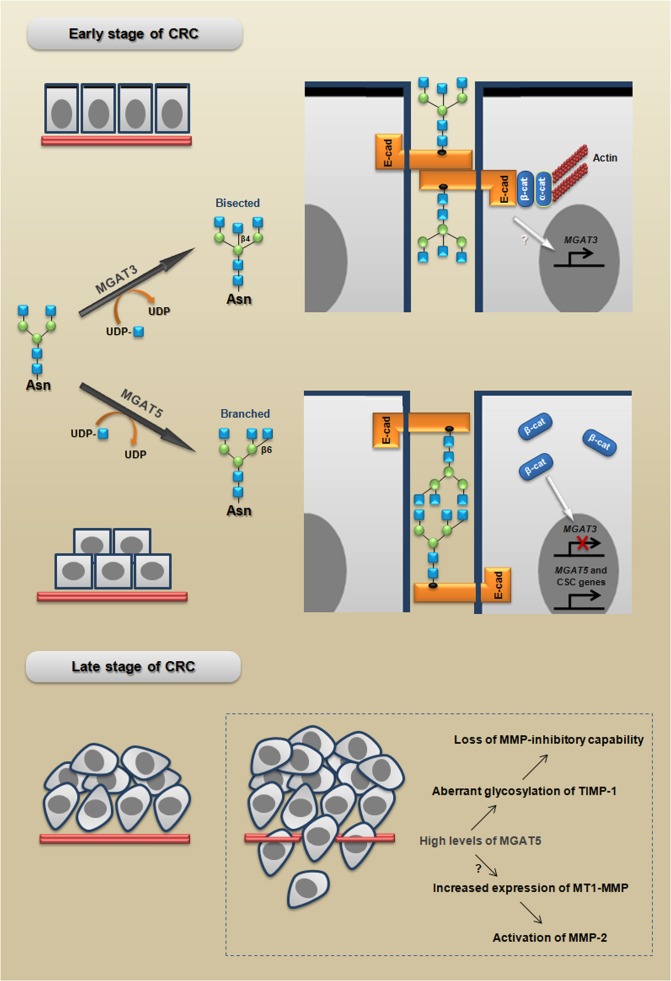
MGAT3 and MGAT5 modulate cell behavior in early and late stages of CRC MGAT3 catalyzes the transfer of GlcNAc from UDP-GlcNAc to the core mannose in a β1,4 linkage, thus generating bisected *N*-glycans, which have been associated with a stable phenotype of E-cadherin-mediated cell-cell adhesion. Adherens junctions formation promote *MGAT3* expression establishing a reciprocal mechanism. In turn, MGAT5 catalyzes the transfer of GlcNAc in a β1,6 linkage, generating branched *N*-glycans, which have been associated with: I) an unstable phenotype of adherens junctions; (II) the inhibition of MGAT3 induced by β-catenin; and (III) increased expression of CSC-related genes. MGAT5 increases also the malignant behavior through both loss of MMP-inhibitory capability and activation of MMP-2. The branched and bisected *N*-glycan scheme is adapted from [120].

An elegant study using DLD-1/Δα cells, a subclone of the human colon carcinoma DLD-1 cell line that lack α-catenin (an actin-binding protein at the AJs) expression, revealed that restoration of the α-catenin gene result in both a rescue of the cortical actin staining pattern (typical of epithelial cells) and a significant increase in MGAT3 activity, suggesting that MGAT3 expression is tightly regulated by cell-cell adhesion via the E-cadherin-catenin complex [57]. Although these results show that the positive regulation of MGAT3 is a consequence of establishment of stable E-cadherin-mediated cell-cell adhesion, it is important to note that increased MGAT3 expression, and consequently bisected *N*-glycans, prolongs E-cadherin turnover on cell surface [58]. Thus, the relationship between bisected *N*-glycans expression and E-cadherin-mediated cell-cell adhesion seems to establish a reciprocal regulatory mechanism (Figure 5, upper panel). Using the same cell line (DLD-1/Δα, which present free cytoplasmic β-catenin) it was demonstrated that that β-catenin *knockdown* (decreased to ~20%) result in a dramatic increase in the expression of MGAT3 and its products. Interestingly, the treatment of these cells with soluble Wnt3a significantly down-regulates MGAT3 expression [59].

#### MGAT5 levels modulate malignant phenotype and stemness

In human colon carcinoma WiDr cells, very interesting results showed that the aberrant glycosylation of TIMP-1 (tissue inhibitor of metalloproteinase-1), through MGAT5 overexpression, increases the malignant behavior and promotes the tumor growth rate [60]. Additionally, using the same cell line it was also demonstrated that MGAT5 triggers the overexpression of MT1-MMP (membrane type-1 matrix metalloproteinase or MMP-14), which increases the invasive potential [61]. Interestingly, on the one hand tumors formed by CRC cells overexpressing MGAT5, injected into NOD/SCID mice, grow faster than the tumors formed by control cells and, on the other hand, tumors formed by injection of cells with MGAT5 suppression (siRNA) grow significantly slower than the tumors formed by control cells [62]. Together, these studies strongly support the pro-malignant role of MGAT5 in CRC; however, more investigations are required to confirm the relevance of these mechanisms during human CRC metastatic processes *in vivo*. Since MGAT5 levels is controlled by the *RAS* oncogene [63], we could speculate that these mechanisms mediated by β1,6-branched *N*-glycans may occur in *RAS*-mutated CRC.

Recently, it was also showed that overexpression of MGAT5 in CRC cells increases the size of population representative of colon cancer stem cells (CSC). In addition, the same study showed that colon cancer cells with stem cell-like properties have a significant increase in the expression of MGAT5 that was accompanied by increased L-PHA (*Phaseolus vulgaris* - leukoagglutinin, a specific lectin for branched tri- and tetra-antennary complex-type *N*-glycans with β1,6-linked *N*-acetylglucosamine) staining [62].

The Figure 5 summarizes the proposed model for how MGAT3 and MGAT5 could participate in both early and late stages of CRC progression.

### α2,6-sialylated lactosamine (Sia6LacNAc) modulates invasiveness and stemness

The upregulation of Sia6LacNAc (see structure in Figure 3) and ST6GAL1 (ST6 beta-galactosamide alpha-2,6-sialyltranferase 1, a sialyltransferase that adds an α2-6-linked sialic acid to the *N*-glycan) are frequently observed in CRC cancers [64, 65]. Curiously, it was shown that the expression of ST6GAL1 in the SW948 human colon cancer cell line (which endogenously lacks ST6GAL1 expression) by one side improves the ability to heal a scratch wound, but on the other side reduces the ability for tumor growth at the subcutaneous site of injection in nude mice [66]. An *in vitro* analysis demonstrated that the suppression of ST6GAL1 reduces the invasiveness and anchorage-independent growth of HT-29 human CRC cells [67]. Moreover, α2-6 sialylation of β1 integrins is increased in colon adenocarcinoma tissues [68] and blocks its adhesion to galectin-3, thus protecting the cells against galectin-3-induced apoptosis in SW48 CRC cells [69]. Recently, a study revealed that the upregulation of ST6GAL1 promotes tumorigenesis and may serve as a regulator of the stem-cell phenotype in CRC cell populations. In addition, the same study showed that ST6GAL1 was highly expressed in induced pluripotent stem (iPS) cells, with no detectable expression in the cells from which iPS cells were derived [70]. It is important to note that phenotypic changes induced by ST6GAL1 are under the control of the *RAS* oncogene [71, 72], which reinforces the notion that its triggered mechanisms may be present during the progression of *RAS*-mutated CRC.

### LacdiNAc termini on *N*-glycans contributes to the maintenance of cell stemness

The LacdiNAc termini (see structure in Figure 3) on *N*-glycans is synthesized by the transfer of terminal *N*-acetylgalactosamine residues to *N*-acetylglucosamine through the actions of β1,4-*N*-acetylgalactosaminyltransferase III (B4GALNT3). Recently, it was demonstrated that B4GALNT3 modifies the *N*-glycans of EGFR with LacdiNAc, and the knockdown of B4GALNT3 inhibits EGF-induced migration and invasion in CRC cell lines. Interestingly, this knockdown suppresses the expression of stem-cell associated markers (OCT4 and NANOG) [73]. LacdiNAc can be found in several glycoproteins, which indicates that B4GALNT3 might regulate the malignant phenotype of CRC cells through other signaling mechanisms; however, more studies are required to elucidate this issue.

### Disruption of fucose synthesis (*de novo* pathway) is involved in cancer progression

Some human colon cancer cells, such as HCT-116, have mutations in *GMDS* (the gene encoding the enzyme GDP-mannose-4,6-dehydratase, which is crucial for the synthesis of the nucleotide sugar donor GDP-fuc via the *de novo* pathway). This mutation impairs fucosylation and results in resistance to TRAIL-induced apoptosis, followed by escape from immune surveillance [74]. Recently, it was demonstrated that the frequency of the *GMDS* mutation in primary CRC tissues is 8.6% (7/81 samples), and in metastatic lesions, this frequency is slightly higher (12.8%, or 5/39 samples) [75]. These findings show that different subtypes of CRC may originate from different mechanisms during the metastatic cascade; reinforcing the idea that tumor signature based on glycan expression can be useful for distinguishing these different subtypes.

## THE EXPRESSION OF GLYCOSYLTRANSFERASES AND N-GLYCANS AS CRC BIOMARKERS

Currently, the correlation between CRC progression and changes in the pattern of expression of *N*-glycans is being considered in search for new biomarkers. Therefore, these markers, which are based on glycosyltransferase and glycan expression, may have several applications, such as the ability to distinguish different disease states, to identify patients who are at high risk for a more aggressive disease and to predict the response or resistance to a particular therapy.

### MGAT5 and β1,6-branched *N*-glycans

In histological sections of colonic tissue, adenomas and high-grade intraepithelial neoplasia were found to show a small increase in L-PHA staining compared to normal colonic epithelium, whereas carcinomas were found to show greatly increased reactivity [76, 77]. Furthermore, L-PHA staining in human colorectal carcinoma sections provides an independent prognostic indicator for tumor recurrence and patient survival and is associated with the presence of lymph node metastases [78]. Corroborating these results, interesting findings showed that the expression of MGAT5 in CRC samples correlates with metastasis and a poor prognosis [79]. Additionally, tumor specimens examined via RT-PCR and compared with the corresponding mucosa from each patient showed that MGAT5 expression is significantly enhanced in colorectal adenomas, carcinomas, and liver metastases [80]. Together, these findings support the potential usefulness of MGAT5 and β1,6-branched *N*-glycans as markers to predict the aggressive phenotype in CRC tumors.

### Bisected *N*-glycans

A mass spectrometry-based analysis concluded that the bi-antennary *N*-glycan levels containing a bisecting β1,4 GlcNAc are decreased in CRC tissues [81]. Similarly, E-PHA (*Phaseolus vulgaris* - erythroagglutinin, a specific lectin for bisected di- and triantennary complex type *N*-glycans with β1,4-linked *N*-acetylglucosamine) staining is more frequently observed in normal compared to tumor epithelia, and the difference is most evident in tumors with microsatellite stability [82]. Comparing the *N*-glycan profiles of membrane proteins in phenotypically different CRC cell lines, it was shown that bisecting β1,4 GlcNAc was expressed only in moderately differentiated cells, whereas its expression was not detected in poorly differentiated cells [83]. These results suggest that the increased expression of bisected *N-*glycans could be related to a low malignant potential.

### B3GNT8 and poly-*N*-acetyllactosamine chains

B3GNT8 is an enzyme that is involved in the biosynthesis of poly-*N*-acetyllactosamine chains (see structure in Figure 3) by transferring GlcNAc to the non-reducing terminus of Galβ1-4GlcNAc on β1,6-branched *N*-glycan [84]. An important study demonstrated that the level of the B3GNT8 transcript was significantly higher in the majority of CRC tissues than in normal tissues [84]. Because CRC cells (HCT15) transfected with B3GNT8 shows an increase in reactivity to L-PHA the authors suggest that this enzyme may be involved in malignancy, by synthesizing polylactosamine on β1,6-branched *N*-glycans.

### Fucosylated *N*-glycans

A study that used oligonucleotide arrays to examine human colonic tissue showed that FUT8 was upregulated only in carcinoma tissue [85]. In contrast, as mentioned previously, the core fucosylation of E-cadherin enhances cell-cell adhesions in human colon carcinoma cells, and mutations in *GMDS* impair fucosylation reactions, contributing to the escape of CRC from immune surveillance [56, 74]. The results linking fucosylated proteins, the expression of FUT8, and CRC are not clear yet, thus more studies are required to elucidate this issue. This particular issue will be discussed further below.

### Sialylated *N*-glycans

In a pioneer study on the detection of altered sialylated *N*-glycan expression in CRC samples, it was demonstrated that the activity of ST6GAL1 is higher in tumor tissues compared to normal counterparts [86]. Corroborating these findings, increased expression of ST6GAL1 was detected by RT-PCR in carcinoma specimens [80]. Beside increased activity and expression of ST6GAL1, the increased expression of α2,6-sialylated sugar chains (see Sia6LacNAc structure in Figure 3) has also been observed in colon cancer specimens, which were detected using SNA (*Sambucus nigra* agglutinin - a specific lectin that binds preferentially to sialic acid attached to a terminal galactose) [87]. Together, these findings support the usefulness of both the activity/expression of ST6GAL1 and the expression of α2,6-sialylated sugar chains detected with SNA as markers to distinguish different disease states of CRC.

Recently, α2,3sialylated type-2 chain structures (common constituents of several cell surface molecules, such as glycolipids, *O*-glycans and *N*-glycans) were found to be predominantly expressed in colorectal tissues associated with malignant transformations, particularly with lymph node metastasis in distal colorectal cancer [88]. Therefore, the combination of markers for different sialylated structures could be an interesting strategy to detect a high metastatic potential in CRC tissues.

### Paucimannose and small high-mannose *N*-glycans

A mass spectrometry-based analysis concluded that the paucimannose *N*-glycans (Man_1-3_Fuc_0-1_GlcNAc_2_) levels are increased in CRC tissues when compared with the control tissues [81]. Recently, an *N*-glycomic profiling of rectal adenomas and carcinomas samples demonstrated that paucimannose *N*-glycans and small high-mannose *N*-glycans were more common in carcinomas than in adenomas. In addition, high levels of paucimannose *N*-glycans in advanced colorectal cancer correlated with poor prognosis [89]. These encouraging results raise the possibility that *N*-glycomic profiling may be a promising tool not only to distinguish different colorectal cancer states but also to identify patients with more aggressive disease.

### Altered glycosylation of plasma proteins

In addition to the *N*-glycosylation patterns observed in colorectal cells, *N*-glycan expression analyses of plasma proteins from patients have also uncovered important information for the development of new biomarkers. Plasma glycoproteomic analyses suggest that the overall levels of plasma protein (complement C3, histidine-rich glycoprotein, and kininogen-1) fucosylation and sialylation, which are recognized by AAL [*Aleuria aurentia* lectin, which binds to fucose-linked (α-1,6) *N*-acetylglucosamine or (α-1,3) *N*-acetyllactosamine] and SNA, respectively, are higher in colorectal cancer and adenoma plasma samples compared to normal plasma controls [90]. Interestingly, this *N*-glycan-based strategy represents a noninvasive alternative for identifying CRC.

## N-GLYCANS BIOSYNTHESIS AS A POTENTIAL THERAPEUTIC TARGET

In view of the essential roles played by structural carbohydrates in diverse cancer-related processes, the manipulation of their expression emerges as a new therapeutic possibility [91, 92]. This possibility is highlighted by the following observations: a) pharmaceutical agents that modulate carbohydrate expression have promising therapeutic potentials for various cancer types [93], and b) some encouraging results have suggested that *N*-glycan biosynthesis is an appealing therapeutic target in cancer [94, 95]. *N*-glycan biosynthesis can be blocked using compounds such as swainsonine or tunicamycin. Swainsonine inhibits the α-mannosidase II enzyme, thereby blocking the formation of complex-type *N*-glycans, and tunicamycin blocks the enzyme that catalyzes the transfer of *N*-acetylglucosamine-1-phosphate from UDP-GlcNAc to dolichol monophosphate, thus inhibiting the formation of a lipid-linked oligosaccharide precursor. Therefore, some studies have used these inhibitors as potential new therapeutic anticancer drugs. For example, swainsonine has been used as a therapeutic anticancer agent in Phase I and II [96, 97]. *In vitro* and *in vivo* studies have shown that swainsonine inhibits the growth of human carcinoma cells [98]. In *in vitro* analyses using CRC cell lines, it was demonstrated that swainsonine reduces 5-fluorouracil (5-FU) tolerance in the multistage resistance [99]. Similar effects were obtained with knockdown of B3GNT8 in a 5-FU-resistant CRC cell line [100]. Additionally, we demonstrated that the combination of swainsonine with cisplatin or irinotecan enhanced their toxicity in undifferentiated HCT-116 CRC cells [101]. We also reported that the inhibition of *N*-linked glycosylation by tunicamycin (low doses) induces functional E-cadherin-mediated cell-cell adhesion, which leads to the inhibition of cell proliferation and the development of a differentiated-like phenotype in undifferentiated HCT-116 CRC cells [37]. Altogether, these results highlight the need to focus more efforts on determining the usefulness of *N*-glycan biosynthesis inhibitors as anticancer agents.

Other important findings have shown that the inhibition of *N*-glycan biosynthesis appears to enhance the effects of radiation. Radiotherapy has been explored for improving the local control and survival of locally advanced rectal cancer [102]. Various chemotherapeutic and biological agents have been used as radiosensitizers in combination with radiotherapy, including 5-FU [103]. In this context, we reported that the inhibition of *N*-glycan biosynthesis radiosensitizes undifferentiated HCT-116 CRC cells [101]. Additionally to the therapeutic effects of radiation, our recent results have shown that resistant progenies derived from irradiated CRC-differentiated cells can lead to increased malignant behaviors, such as migration and invasion [104]; therefore, the use of pharmacological inhibitors of *N*-glycan biosynthesis could be useful for reducing the malignant potential of these progenies. Interestingly, it was demonstrated that the increase of integrin β1 sialylation by ST6GAL1 is particularly involved in the radiation-mediated cell migration of CRC cell lines [105].

It is well known that an *N*-glycosylation blockade disturbs protein folding in the endoplasmic reticulum (ER), resulting in ER stress. The adverse effects of accumulating unfolded proteins activates a set of signaling pathways that are termed the unfolded protein response (UPR). Signaling initiated from the UPR actively participates in autophagy and both the intrinsic and extrinsic apoptosis pathways, and under acute prolonged ER stress, apoptosis is triggered [106, 107]. Previous studies showed that even *N*-glycan biosynthesis inhibitors, such as tunicamycin, are not potential therapeutic agents because of their narrow efficacy window; however, a compound with similar biological effects but with a broader therapeutic window could be useful, such as for the chemo- or radiosensitization of cancer cells [94].

In addition to *N*-glycan biosynthesis inhibitors, cell-permeable fluorinated analogs of monosaccharides represent a new approach for remodeling the cell glycome by serving as important tools to dissect the role of glycan modifications within complex biological systems, such as cancer [92]. These molecules are processed by monosaccharide salvage pathways to generate glycosyltransferase inhibitors intracellularly. Therefore, fluorinated analogs of sialic acid could be useful for reducing both the sialic acid-mediated adhesive potential (e.g., decreasing Sialyl Lewis X on the surface glycoproteins of tumors) and Sia6LacNAc expression. However, the real potential therapeutic applications of this strategy remain unclear and justify further investigation, particularly in CRC.

Interestingly, other anticancer drugs affect the expression levels of some glycosyltransferases. For instance, DNA hypomethylation of CRC cells that is induced by treatment with 5-azacytidine (a demethylating agent that has shown significant clinical benefits in clinical trials) enhances the expression levels of FUT8 [108]. Additionally, treatment with mesalamine (an anti-inflammatory drug used to treat ulcerative colitis) increases MGAT3 expression in mice intestinal polyps (which normally show a low expression of MGAT3) [109]. Together, these results show that both the manipulation of the epigenome and treatment with anti-inflammatory drugs represent alternative strategies to remodel the glycome in CRC cells.

Recently, robust evidence has shown that the concentration of oxygen can influence the *N*-glycan profile in surrounding cancer cells, such as endothelial cells. It was demonstrated that hypoxia by one side increases the amounts of β1,6-branched *N*-glycans and, on the other side, reduces α2-6 sialylation levels. More importantly, high levels of β1,6-branched *N*-glycans confer resistance to anti-VEGF therapy, and interruption of β1,6-branching in endothelial cells converts refractory into anti-VEGF-sensitive tumors. In addition, high levels of α2-6 sialylation feature anti-VEGF-sensitive tumors, and the elimination of α2-6-linked sialic acid confers resistance to anti-VEGF treatment [110]. Although these results strongly support the role of β1,6-branched *N*-glycans and α2-6 sialylation for antiangiogenic therapy (used in the treatment of CRC), more investigations are required to confirm the relevance of these mechanisms in CRC tissues.

## CONCLUSIONS AND FUTURE DIRECTIONS

Despite multiple therapeutic advances, such as molecular targeted therapy, the prognosis for patients with metastatic CRC remains poor, with a median overall survival of approximately 20 months [111]. This highlights the need to research new targets and therapeutic strategies to overcome CRC. As here discussed, it is evident that aberrant glycosylation plays crucial roles during different stages of CRC. Although it is not yet defined whether *N*-glycan biosynthesis could be considered a new target for CRC therapy, the data gathered here raise this possibility. In CRC, several cellular mechanisms related to activation of the EMT program, such as the acquisition of migratory and invasive phenotypes, appear to be mediated by *N*-glycan. Therefore, therapies targeting *N*-glycan biosynthesis could be useful for blocking EMT-related cellular features.

In recent years, robust evidence has shown that EMT generates cells with the properties of stem cells [112]. Additionally, it was also demonstrated that drug treatment (e.g., oxaliplatin) of colon cancer results in an enrichment of cancer stem cells (CSC), thus boosting the abundance of these cells by more than 10 times [113]. It is well known that the therapeutic strategies to target CSC must overcome the challenges of specificity before they can be translated into the clinic [114]. Therefore, the *N*-glycan expression pattern could be used to improve the CSC signature. For instance, cell surface expression of the AC133 epitope on CD133, which is a CSC-associated marker, is affected by CD133 *N*-glycans [115].

**Figure 6:**
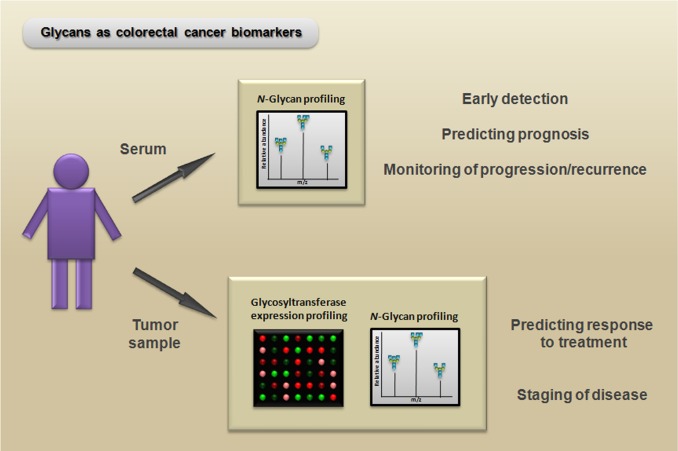
Potential biomarker and therapeutic applications of *N*-glycans Serum *N*-glycan profiling (e.g., via mass spectrometry) of patients with colorectal cancer can be useful as a noninvasive method for early detection, predicting prognosis, and monitoring progression/recurrence. Moreover, glycosyltransferase expression profiling (e.g., via PCR array) paired with *N*-glycan profiling of colorectal cancer samples can reveal important information for predicting the response to treatment and can aid in distinguishing between different disease states.

In regard to improving the diagnosis of CRC, the use of biomarkers based on *N*-glycan and glycosyltransferase expression may contribute to the development of more sensitive and reliable techniques for early detection of the disease. Recently, a serum *N*-glycan profiling study using DNA sequencer-assisted/fluorophore-assisted carbohydrate electrophoresis (DSA-FACE) demonstrated that *N*-glycan marker-based diagnostic models represent new, valuable, and noninvasive alternatives for identifying CRC [116]. Because glycosylation is a complex system, the development of biomarkers based on *N*-glycans is particularly challenging; however, this field is enhancing simultaneously with the advancement of analytical technologies for elucidating carbohydrate structures. Protocols using liquid chromatography (LC) paired with mass spectrometry (MS) have been used for large-scale analyses of *N*-glycan expression, and they even have the ability to distinguish structural isomers (see [117] for a review of the applications of these techniques for glycomic and glycoproteomic biomarker discovery). Moreover, future studies to determine the expression pattern of glycosyltransferases via PCR arrays or microarrays paired with *N*-glycan profiles could be useful for many applications, such as improving the prediction of both the treatment outcomes and the stage of the disease (Figure 6).

In addition to LC paired with MS, lectin microarrays represent an alternative approach to glycan analysis by a simple procedure, since this method enables direct analysis of samples containing glycoproteins, in the absence of automated systems and without the need for liberation of glycans from their core proteins [118]. Other useful approach to identifcation of glycoproteins with potential roles in cancer initiation and progression is the enrichment through lectin affinity chromatography followed by proteomic analysis.

It is important to highlight that some results discussed here, which use the current model systems (e.g. immunocompromised mice or long-ago established cell lines) have several limitations, since the inflammatory microenvironment plays a crucial role during the carcinogenic process. However, alternative approaches such as patient-derived xenografts and immunocompetent mouse models are thought to offer more reliable data and will be useful to unravel the glycocode with significance in CRC.

Recently, very interesting findings suggested that miRNA are a crucial regulator of the human glycome [119]; therefore, the identification of miRNA-dependent changes of *N*-glycosylation could be also useful to unravel the glycocode in CRC. Moreover, the integration of changes in glycogenes expression/activity and the mutational landscape of CRC [e.g., from initiatives like The Cancer Genome Atlas (TCGA)] may contribute to the understanding how such changes interact to drive the CRC and, consequently, to develop both more specific treatments and more reliable strategies for early detection. Furthermore, glycogenes themselves can be interrogated in large data sets, which include cancer cell lines and tumors [e.g., Cancer Cell Line Encyclopedia (CCLE) and Catalogue of Somatic Mutations in Cancer (COSMIC)] to analyze changes in expression between normal versus tumor and/or tumor grade/stage, providing a great resource for validation of experimental findings.

Despite the encouraging results presented and discussed here, several open questions show that many pieces must be connected to solve the puzzle generated by the advances in the field of glycobiology in CRC. For example, is there a relationship between the overexpression of FUT8 and mutations in *GMDS* during CRC progression? Could the overexpression of FUT8 be explained by a feedback mechanism? Only future and more detailed studies will answer these questions and determine the actual pathological relevance of *N*-glycans to exploit their potential biomarker and therapeutic applications, particularly in CRC.

## Abbreviations

AKT1: v-akt murine thymoma viral oncogene homolog 1
APC: adenomatous polyposis coli
BAX: BCL2-associated X protein
BCL2: B-cell CLL/lymphoma 2
BRAF: v-raf murine sarcoma viral oncogene homolog B
DNA: deoxyribonucleic acid
EGF: epidermal growth factor
EGFR: epidermal growth factor receptor
ERK: extracellular-signal-regulated kinases
FGFR: fibroblast growth factor receptor
GDP-Fuc: guanosine diphosphate fucose
GSK3-β: glycogen synthase kinase-3 beta
IGF-I: insulin-like growth factor 1
IGF-IR: insulin-like growth factor 1 receptor
IGFR: insulin-like growth factor 1 receptor
IR: insulin receptor
KRAS: Kirsten rat sarcoma viral oncogene homolog
MAPK: mitogen-activated protein kinases
MMP: matrix metalloproteinase
MTOR: mechanistic target of rapamycin
NOD/SCID: nonobese diabetic/severe combined immunodeficiency
PDGFR: platelet-derived growth factor receptor
PI3K: phosphatidylinositol 3-kinase subunits gene family
PTEN: phosphatase and tensin homolog
BRAF: B-Raf proto-oncogene - serine/threonine kinase
RAS: RAS type family GTPases
RT-PCR: reverse transcription polymerase chain reaction
SMAD4: SMAD family member 4
TCF/LEF: T-cell factor/ lymphoid enhancing factor
TGF-β: transforming growth factor beta
TP53: tumor protein p53
TRAIL: TNF (tumor necrosis factor)-related apoptosis-inducing ligand
UDP-GlcNAc: uridine diphosphate N-acetylglucosamine
VEGF: vascular endothelial growth factor
Wnt: wingless-type MMTV integration site family.
